# Medico-Legal Evidence in Cases of Insanity

**Published:** 1853-05

**Authors:** D. Miller

**Affiliations:** Chicago


THE NORTH-WESTERN

MEDICAL AND SURGICAL JOURNAL.

NEW SERIES.

VOL. II. MAY, 1853. No. 1.

### ORIGINAL COMMUNICATIONS.

ARTICLE I.—*Medico-Legal Evidence in Cases of Insanity.* By D. Miller, M.D., Chicago.

The importance of medical evidence in cases of insanity, and the liability of physicians to be called upon, at any time, for an opinion, would seem to be sufficient inducements to study thoroughly, not only the subject of insanity, but also the opinions and decisions in courts of justice. Should other incentives be necessary, they exist in the instances, not of infrequent occurrence, of mortification, not to say disgrace, to which the medical witness is subjected in courts of law. This is not, however, always the result of ignorance or inconsistency on the part of the witness; for frequently he is embarrassed from the fact, that "there exists no settled, no uniform, no fixed, no unerring principles of jurisprudence, or *legal tests*, in regard to questions of criminal or civil insanity." And, again, he is frequently required to give an opinion upon facts presented through a third party, and these of a vague, uncertain, and many times contradictory character.

In that valuable work, the *American Journal of Insanity*, for April, is an abstract of a Lecture on Medico-Legal Evidence in Cases of Insanity, by F. Winslow, M.D., from which we take the following "*Legal Criteria*":—

"Analyzing with great care the *dicta* of the judges, it would appear that the courts of law have, upon different occasions, admitted the following conditions of mind as evidence of insane and legal irresponsibility:—

"1. An absolute dispossession, by disease, of the free and natural agency of the mind; partial insanity being no excuse for crime.

"2. The existence of a delusion, the criminal act being the immediate and direct result of the morbid idea; the proof of the presence of a delusion having no positive and clear connection with the alleged crime, not being legal insanity, and no evidence of the existence of irresponsibility.

"3. A consciousness of offending against the laws of God and man—in other words, a knowledge of good and evil.

"4. A knowledge of right and wrong—lawful or unlawful—the presence or absence of motive."

It would seem that the above "dicta" would be clear to the apprehension of the mind, and of easy application; but without looking farther, we gather from the abstract of the lecture before us, the following conflicting opinions and decisions:—

"If the delusion were only partial, the party accused was *equally liable* with a person of sane mind. If the accused killed another in self-defence, he would be entitled to an acquittal; but, if the crime was committed for any supposed injury, he would then be liable to the punishment awarded by law to his crimes."

This repudiates *partial insanity* as a plea in extenuation of crime, or as an exemption from punishment. According to the 64th Article of the French Penal Code, no person, whilst insane is considered responsible for a criminal act. The Advocate-General of France insisted that "total insanity can alone extricate the criminal from the penalties of the laws;" and referred to the opinions of Lord Hale in confirmation of the position. From which, however, the medical jurists of France dissented; and M. Georget expressed his astonishment at the "dicta" of Lord Hale. Again, "partial insanity" has been held as a valid plea, under the French code.

"Partial insanity is defined to be, 'A mind in an unsound state—not unsound upon one point only, and sound in all other respect, but that this unsoundness manifests itself principally with reference to some particular object or person.'" This definition, we think, very properly makes a distinction between "partial insanity" and "monomania," which, however, have been considered nearly equivalent terms by medical and legal authorities.

*The Test of Delusion.*—"The true test of insanity," says Sir John Nicoll, "I take to be, absence or presence of what, used in a certain sense, is compressible in a single term, viz., *Delusion;*" which he defines to be, "A belief of facts, which no rational reason would have believed." And Lord Denman, "To say a man was irresponsible, without positive proof of any act to show that he was laboring under some delusion, seemed to him to be a presumption of knowledge which none but the great Creator could himself possess." And Lord Brougham defines a delusion to be, "A belief of things as realities which exist only in the imagination of the patient." This definition is subject to the objection, that it involves exclusively the actions of the *healthy* imagination. A better definition, as we believe, would seem to be, "A delusion is a belief in the existence of a something extravagant, which has in reality no existence, except in the diseased imagination of the party, and the absurdity of which he cannot perceive, and out of which he cannot be reasoned." Legal authorities have considered *delusion* to be the legal test of insanity; and yet, in the case of Overston, Justice Mould altogether repudiated the test; and in the celebrated Bainbridge case, Lord Campbell maintained that "*mania may exist without delusion.*" "Need I advance one argument in corroboration of Lord Campbell's dictum, or in opposition to the dogmatic and bold assertion of Lord Denman?"—We all admit—it is the result of the collective experience of all competent to give an opinion upon the matter—that positive, dangerous, and irresponsible insanity, may exist, and often does exist, without any *manifested* delusive impression, or *appreciable* hallucination.

"*The Presence or Absence of a Motive.*—The next legal test that presents itself for my consideration is, the presence or absence of a *motive* for the commission of the crime. Dr. Prichard observes, 'The act of homicidal insanity is different in its nature and moral causes from that of murder. Men never commit crimes without some motive; the inducement which leads them to an atrocious act is of a kind which other men can appreciate and understand, though they do not sympathise with them. Jealousy, hatred, revenge, excite some; others are moved by the desire of plunder—of getting possession of money or property. The act of a madman is for the most part without motive."

This principle of Dr. Prichard seems not only unsafe but unphilosophical. On the trial of Francis for shooting the Queen, this plea was urged in favor of the prisoner; but, after reviewing the case, the Solicitor-General lays down the following principle:—"The insane are often impelled to the commission of acts of violence and murder by the same motives, feelings, and passions, that influence and regulate the conduct of sound, healthy, and rational minds."

"*The consciousness that the act was a criminal one, and one in opposition to the laws of God and man.*—It has been proposed, that the question of legal responsibility should be determined by the fact, whether the party, when he committed the offence, knew that he was acting in opposition to those generally-received and recognised moral obligations which are supposed to govern and influence sane rational, and christian minds. The question put to the jury, to use the language of one our most distinguished ex-chancellors, is "Was the prisoner conscious that he was committing a crime against the laws of God and nature?"

"Imagine a person arranged for the commission of a capital crime. The plea of insanity is urged in his defence. In expounding the law, the judge informs the jury, that the question of responsibility in connexion with insanity rests upon the fact whether the prisoner had at the time a consciousness of his having deviated from the law of God. Was he sensible of this, or was he not?—If so, he is to be considered amendable to justice, and must expiate his crime upon the gallows, I can conceive, that after such an exposition of the law, the prisoner making a declaration of his being by virtue of his atheistical principles *placed beyond the jurisdiction of such a test*, he could not morally, legally, or logically be considered to be conscious of violating what in reality he never believed to exist. I will admit that this may be considered to be an extreme hypothesis. I merely cite it with the view of establishing my position, that there is no legal test yet propounded applicable, or which could be indiscriminately applied, to all criminal cases of insanity."

*The test of Right and Wrong psychologically analyzed.*—Chief Justice Tyndall held that, we are to determine the presence of legal responsibility, by applying the test of a *knowledge* of right and wrong,—and Lord Mansfield, "If a man was deprived of all power of reasoning, so as not to be able to distinguish whether it was right or wrong, to commit the most wicked act, or the most innocent transaction, he could not certainly commit an act against the law!"

It has been a question with metaphysicians, whether, *abstract-*

*ly* considered, there are conditions or states to which terms "right" and "wrong" can, with strict philosophical precision be applied· Drs. Hutchinson, Brown, and others, hold the following:--That there is no right or wrong, merit or demerit, *existing independently of the agents, who are virtuous or vicious.*

"The *right* of *to-day*, in matters of theology, philosophy, and science, may be the *wrong* of *to-morrow;* and what is now "lawful" may, in the course of a short parliamentary session, be made illegal by the introduction of new statutes! Analyzing this much-eulogized legal test as metaphysicians, as medical philosophers, and as men of the world, are we not compelled to pronounce it to be worthless, and unsusceptible of any practical application?"

"*The test of Right and Wrong pathologically considered.* —Considering this legal test of criminality apart altogether from the metaphysical objections to which it is amenable, I maintain, that it never can be safely depended upon, in all cases of insanity. It is a notorious fact—a matter of every-day occurrence, and in accordance with the experience of all men of observation, that the insane—the positively and undeniably insane—like many rational persons, often

"Know the right, and yet the wrong pursue."

"When Martin set York Minster on fire, a conversation took place among the inmates of a neighboring lunatic asylum, having reference to this general topic of remark and discussion. The question argued was whether Martin would suffer the extreme penalty of the law for his crime. Various were the opinions expressed. In the midst of the conversation, one patient, apparently as mad as the rest, exclaimed, "He (Martin) will not be hanged." "For what reason?" interrupted many voices. "They cannot hang him," replied the lunatic, "*he is one of ourselves.*" Of what value is this legal test if applied to such cases? Before this can be recognized as a safe standard, it will be necessary for British jurists to lay down for their *own* guidance certain fixed and unalterable principles of jurisprudence. Is it not a notorious fact that on apparently clear and well-recognized points, lawyers of eminence have arrived at the most opposite conclusions? One court reverses the judgment of an inferior tribunal, and one distinguished jurist overrules the decision of his predecessor. As long as able judges differ among themselves upon what may be termed *first principles of law*, it will be unresonable to expect that physicians should prostrate themselves before the legal test which I have been analyzing."

We have extended these extracts much beyond intention, and would close by recommending to the Medical Profession the *American Journal of Insanity*, published quarterly at Utica N. Y. at one dollar and fifty cents a year, in advance.

